# Supplementary material for: Food insecurity and COVID-19-related experiences among people with HIV: A mixed methods analysis and conceptual framework
Source: PLoS One. 2026 Feb 12;21(2):e0342667. doi: 10.1371/journal.pone.0342667 (PMC12900320; doi:10.1371/journal.pone.0342667)
Supplement: S2 File — (DOCX) [file pone.0342667.s003.docx]

**S2 File. Qualitative codebook**

March 24, 2025

1. **Food insecurity**
   1. Overall perceptions of degree of food insecurity
   2. Food access:
      1. Inability to have services delivered
      2. Inaccessibility of grocery store:
         1. Physical barriers: age, physical health
         2. Mental health barriers: stress and anxiety related to going out, obtaining food/groceries, COVID-19 exposure
         3. Logistical barriers: Need for public transit
      3. Cost of food
   3. Perceived stigma or guilt:
      1. HIV stigma
      2. Immigration status
      3. Guilt about accessing food assistance
   4. COVID impact on food assistance:
      1. Increased demand
      2. Variability of available food
2. **Housing**
   1. Type of housing
   2. Number of people in the house
   3. Housing instability
   4. Housing as COVID risk
3. **Competing obligations**
   1. Gainful employment/essential work role
   2. Caregiving obligations
   3. Work/family as COVID risk
4. **Challenges to following COVID-19 guidelines**
   1. Crowded public spaces (public transport, stores)
   2. Inaccessibility of face masks
   3. Other people not following guidelines
