# Supplementary material for: Food insecurity and COVID-19-related experiences among people with HIV: A mixed methods analysis and conceptual framework
Source: PLoS One. 2026 Feb 12;21(2):e0342667. doi: 10.1371/journal.pone.0342667 (PMC12900320; doi:10.1371/journal.pone.0342667)
Supplement: Attachment — Submitted filename: Reviewer Comments_PLOSONE.docx [file pone.0342667.s004.docx]

**Reviewer Comments – Major Revision Recommended**

**General Assessment**

The manuscript addresses an important and timely topic by examining food insecurity among people with HIV (PWH) during the COVID-19 pandemic using a mixed-methods approach. The study provides valuable insights into multilevel drivers of food insecurity and integrates both quantitative and qualitative evidence from two urban clinics in the northeastern United States.
However, the paper requires substantial revisions to improve methodological transparency, data validity, and overall clarity. Several critical aspects of sampling, measurement, statistical analysis, and qualitative reporting need strengthening before the paper can meet *PLOS ONE* standards for rigor and reproducibility.

**1. Sampling, Inclusion Criteria, and Generalizability**

The study draws participants from an ongoing research collaboration on tobacco use among PWH, with recruitment based on documented tobacco use in the electronic medical record. This sampling frame introduces **potential selection bias**, as tobacco users may differ from non-users in socioeconomic, behavioral, and health characteristics that are also associated with food insecurity.
**Recommendation:** Provide a detailed explanation of inclusion and exclusion criteria. Explicitly state whether all participants were current tobacco users. Discuss the implications of this sampling design for the generalizability of results, and include this limitation in the Discussion section.

**2. Response Rate and Non-Response Bias**

Of 719 individuals contacted, only 283 completed the survey (~39% response rate). This relatively low participation rate raises concerns about non-response bias, particularly if respondents differ systematically from those who did not participate.
**Recommendation:** If possible, compare key demographic or clinical characteristics between respondents and non-respondents using available electronic medical record data (e.g., age, gender, race, CD4 count, last clinic visit). If such data are unavailable, at least discuss the direction and magnitude of possible bias and its potential effect on prevalence estimates and regression outcomes.

**3. Measurement of Food Insecurity**

Food insecurity was assessed using a single self-reported item about the perceived impact of COVID-19 on participants’ ability to obtain or pay for food. While this item provides contextual information, it does **not represent a validated measure** such as the USDA Household Food Security Survey Module or the HFIAS. Consequently, prevalence estimates and comparisons with other studies may be unreliable.
**Recommendation:**

- Justify the choice of a single-item measure and acknowledge its limitations.
- Conduct sensitivity analyses using alternative cut-offs if applicable.
- Expand the Discussion to note that the measure captures *perceived* rather than *experienced* food insecurity.

**4. Study Design and Causality**

Because the study uses a **cross-sectional design**, it cannot establish causal relationships between COVID-related disruptions and food insecurity. The conceptual model presented in the Discussion implies bidirectional relationships, but these are not empirically tested.
**Recommendation:** Clearly distinguish between observed associations and hypothesized causal mechanisms. Rephrase causal language (e.g., “led to,” “caused”) into non-causal wording such as “was associated with.” Include this clarification in both the Abstract and Discussion.

**5. Statistical Analysis and Model Transparency**

The description of the statistical procedures lacks detail. It is unclear how variables were selected for the multivariable model, whether multicollinearity was assessed, how missing data were handled, and whether model assumptions were tested. The manuscript also does not provide full regression outputs (odds ratios, 95% confidence intervals, p-values for all predictors).
**Recommendation:**

- Present a complete multivariable logistic regression table.
- Specify the model-building strategy (a priori, stepwise, or significance-based).
- Report diagnostic statistics (e.g., VIF for multicollinearity, Hosmer–Lemeshow test, AUC).
- Describe how missing data were treated (listwise deletion, imputation, etc.).
- Conduct sensitivity analyses to test robustness (e.g., stratified by clinic site or alternate food insecurity thresholds).

**6. Handling of Missing Data and Data Availability**

Although the manuscript mentions minor missing data, there is no clear statement on how these were managed. Furthermore, *PLOS ONE* requires a transparent **Data Availability Statement**. The current version only states that data are “available upon request,” which does not comply with journal policy.
**Recommendation:**

- Describe how missing values were handled analytically.
- Deposit de-identified data and analysis code in a recognized repository (e.g., Dryad, Zenodo) or explain ethical restrictions preventing public release.
- Provide the repository link or DOI in the Data Availability section.

**7. Ethical Approval and Informed Consent**

The manuscript indicates approval by the Yale and SUNY Downstate Institutional Review Boards and that participants gave verbal consent, but the **protocol approval numbers** are not provided.
**Recommendation:**
Include IRB approval numbers for each institution and explain why verbal consent was used (e.g., due to remote data collection during the pandemic). Clarify how confidentiality and data protection were ensured during telephone interviews.

**8. Funding Disclosure and Role of the Sponsor**

Funding is acknowledged from NIH grants (P30MH062294; R01CA243910), but it is unclear which authors received these awards and whether the funders had any role in the study’s design, analysis, or publication decision.
**Recommendation:**
Provide full grant numbers, specify award recipients, and explicitly state the funders’ role in accordance with *PLOS ONE* disclosure requirements.

**9. Qualitative Analysis: Rigor and Integration**

The qualitative component lacks sufficient methodological detail. The process for coding, the number of coders, how inter-coder reliability or consensus was achieved, and whether data saturation was reached are not clearly described. The integration between quantitative and qualitative results is also limited.
**Recommendation:**

- Elaborate on the coding framework, analytical approach (inductive/deductive), and verification steps.
- Include exemplar quotations supporting each theme in a table or appendix.
- Explain how qualitative and quantitative findings were triangulated to construct the conceptual model.

**10. Results Presentation and Figures/Tables**

Tables and figures require additional clarity. Denominators, missing data counts, and confidence intervals should be consistently reported. The conceptual framework figure should include clear labeling and be explicitly referenced in the text.
**Recommendation:**

- Ensure all tables include n values, percentages, and missing cases.
- Add footnotes describing statistical tests used.
- Improve the figure’s readability and provide a descriptive caption explaining each component.

**11. Writing Quality and Structure**

While the paper is generally well organized, some sentences are awkwardly phrased or repetitive. The manuscript would benefit from professional English language editing to improve flow, grammar, and precision.
**Recommendation:**

- Revise for conciseness and clarity, especially in the Abstract and Discussion.
- Consider editing by a native English-speaking academic editor to ensure alignment with *PLOS ONE* style.

**Summary Recommendation**

The study contributes important evidence about the intersection of HIV, food insecurity, and pandemic-related stressors. However, before publication, the authors must substantially enhance methodological transparency and analytic rigor.
If the above revisions are adequately addressed—particularly concerning sampling clarification, statistical reporting, data availability, and ethical documentation—the manuscript could make a valuable contribution to the literature on health inequities and food security among vulnerable populations.
