# Supplementary material for: Food insecurity and COVID-19-related experiences among people with HIV: A mixed methods analysis and conceptual framework
Source: PLoS One. 2026 Feb 12;21(2):e0342667. doi: 10.1371/journal.pone.0342667 (PMC12900320; doi:10.1371/journal.pone.0342667)
Supplement: Attachment — Submitted filename: Results of a manuscript review entitled_PLOSONE.docx [file pone.0342667.s006.docx]

**Results of a manuscript review entitled**

**"Food insecurity and COVID-19-related experiences among people with HIV: A mixed methods analysis and conceptual framework"**

I have carefully reviewed and verified the revisions made in response to the recommendations provided. The author has addressed the comments thoroughly, and I find the revised manuscript acceptable.
